# Data on characterization of dredging sediment of Safi harbour – Morocco

**DOI:** 10.1016/j.dib.2019.104853

**Published:** 2019-11-22

**Authors:** Ahmed Loudini, Mounsif Ibnoussina, Ali Limam, Azzouz Kchikach, Filipe Duarte González

**Affiliations:** a Faculty of Science Semlalia, Boulevard Prince Moulay Abdellah, Marrakech, Morocco; b Institut national des sciences appliquées de Lyon, 20 Avenue Einstein, 69100 Villeurbanne, France; c Lusíada University of Lisbon, R. da Junqueira 188-198, 1349-001 Lisboa, Portugal

**Keywords:** Dredging sediment, Geotechnical characteristic, Chemical characteristic, Mineralogical characteristic

## Abstract

This data article reports the geotechnical (the water content, the specific density, the Atterberg limits, the methylene blue value and the grain size distribution), chemical (the content of organic matter and calcium carbonate) and mineralogical characteristics of sediments dredged in Safi harbour (Morocco). Samples are collected in seven point (P1, P2, P3, P4, P5, P6 and P7) of two zones of port (the channel zone and the basin zone), placed in opaque drums and kept in a cold room protected from light. Then the homogeneous samples are mixed.

We note S1 the mixture of P1, P2 and P3 correspond to the basin zone of the port and S2 the mixture of P4, P5, P6 and P6 correspond to the channel zone of the port.

**Table:** Specifications Table

Subject	Civil and Structural Engineering
Specific subject area	Building materiel
Type of data	Table Figure
How data were acquired	The water content is measured using the French standard NF P 94-050. The particle-size-distribution is measured according to the French standards NF P 94-056 and NF P 94-057. The density is measured following the AFNOR standard NF P 94-054. The test of Atterberg limits is carried out in accordance with the standard NF P 94-051. The value of the methylene test is realized by following the standard NF P 94-068. The determination of the organic content is measured according to AFNOR standards NF P 94-055 and XP P 94-047. The analysis of the content of calcium carbonate is realized according to the principles of the standard NF P 94-048. The mineralogical analyses are performed using the X-ray powders diffraction technique. The collected data are processed by a software, which gives directly the spectra.
Data format	Raw and analysis
Parameters for data collection	Samples are collected by a diver and placed in opaque 20 Littre drums and then they are kept in a cold room protected from light. On each container, we mark the following information: The identification code of the sampling station, the date, the time and depth.
Description of data collection	Sampling concerns two areas of the port: zone 1 and zone 2 which correspond respectively to the channel of access and the basins of the port of trade. In a first step, sediment samples were collected in 7 points (4 points in zone 1 and 3 point in zone 2). Then the samples were homogenized manually in order to have a single representative sample for each zone. We note S1 for the mixture of P1, P2 and P3 corresponding to the zone 1 and S2 for the mixture between P4, P5, P6 and P7 corresponding to the zone 2.
Data source location	SAFI HARBOUR, SAFI, MORROCO (32.312642, −9.252548)
Data accessibility	Data incorporated within this article.

**Table:** 

**Value of the Data** • The data represents the geotechnical, chemical and mineralogical characteristics of sediments dredged in Safi harbour (Morocco) in order to valorise them in civil engineering works. • These data can be beneficial to all researchers works on valorisation of sediment dredging in port • These data can initiate port users in developing countries to the possibility of valorise dredged sediments instead of dumping them into the sea.

## 1 Data description

The dataset in this article describes geotechnical, chemical and mineralogical characteristic of sediment dredged in Safi harbour.

In Table 1, we present the evolution of water content after drying for one month and the density specific, of five sampling of sediment S1 and S2 dredged in Safi harbour.

**Table 1:** Evolution of water content After drying and Density specific of safi harbour sediment.

Samples			1	2	3	4	5	Average	Median	Mean deviation	standard deviation
S1	Density specific t/m^3^		2,54	2,55	2,58	2,58	2,55	2,56	2,55	0,0160	0,8410
	Evolution of water content After drying (%)	Initial	136	124	100	115	125	120	124	10,00	37,80
		02 days	100	94	79	87	95	91	94	6,40	28,72
		07 days	75	70	59	66	71	68	70	4,56	21,57
		15 days	44	41	35	38	42	40	41	2,80	12,60
		21 days	22	20	15	18	20	19	20	2,00	5,98
		30 days	13	12	8	11	11	11	11	1,20	3,50
S2	Density specific t/m^3^		2,6	2,59	2,59	2,63	2,59	2,60	2,59	0,0120	0,0155
	Evolution of water content After drying (%)	Initial	29	36	34	30	36	33	34	2,80	2,97
		02 days	20	30	28	24	28	26	28	3,20	3,58
		07 days	15	23	22	19	21	20	21	2,40	2,83
		15 days	10	15	14	12	14	13	14	1,60	1,79
		21 days	7	10	10	9	9	9	9	0,80	1,10
		30 days	5	8	8	7	7	7	7	0,80	1,10

In Table 2, we present the particular size distribution of three sampling of sediment S1 and S2 dredged in Safi harbour.

**Table 2:** The particular size distribution of sediment S1 and S2 dredged in Safi harbour.

Samples			1	2	3	Average	Median	Mean deviation	standard deviation
S1	Particle size distribution (%)	Ø < 2 (μm)	8,5	10,5	8	9	8,5	1,00	1,08
		Ø < 20 (μm)	39,1	44,5	37,9	40,5	39,1	2,67	2,87
		Ø < 32 (μm)	40,8	47,3	41,1	43	41,1	2,82	3,00
		Ø < 63 (μm)	58	60	53	57	58	2,67	2,94
		Ø < 80 (μm)	60	65	58	61	60	2,67	2,94
		Ø < 160 (μm)	70,5	75	67,5	71	70,5	2,67	3,08
		Ø < 315 (μm)	90	93	90	91	90	1,33	1,41
		Ø < 400 (μm)	93	96	93	94	93	1,33	1,41
		Ø < 500 (μm)	94,5	97	95	95,5	95	1,00	1,08
		Ø < 630 (μm)	95	98,5	96	96,5	96	1,33	1,47
		Ø < 1250 (μm)	98,6	100	99	99,2	99	0,53	0,59
		Ø < 2000 (μm)	100	100	100	100	100	0,00	0,00
	Hazen Coefficients	Uniformity coefficient	20.96						
		Curvature coefficient	0.78						
	Classification according to the triangle of soil texture USDA		loam						
S2	Particle size distribution (%)	Ø < 2 (μm)	0	0	0	0	0	0,00	0,00
		Ø < 20 (μm)	0	0	0	0	0	0,00	0,00
		Ø < 32 (μm)	0	0	0	0	0	0,00	0,00
		Ø < 63 (μm)	0,5	0	1	0,5	0,5	0,33	0,41
		Ø < 80 (μm)	1	0	4	1,7	1	1,56	1,70
		Ø < 160 (μm)	5	3	10	6	5	2,67	2,94
		Ø < 315 (μm)	75	77	85	79	77	4,00	4,32
		Ø < 400 (μm)	90	91	93,5	91,5	91	1,33	1,47
		Ø < 500 (μm)	96,5	96,5	98	97	96,5	0,67	0,71
		Ø < 630 (μm)	98	99	100	99	99	0,67	0,82
		Ø < 1250 (μm)	100	100	100	100	100	0,00	0,00
		Ø < 2000 (μm)	100	100	100	100	100	0,00	0,00
	Hazen Coefficients	Uniformity coefficient	1.65						
		Curvature coefficient	0.93						
	Classification according to the triangle of soil texture USDA		Sand						

We present in Table 3 the cleanliness of sediment S1, S2 dredged in Safi harbour using the methylene blue test supplements the sand equivalent, and Atterberg limits test (five samples for each test).

**Table 3:** Cleanliness of sediment S1 and S2 dredged in Safi harbour.

Samples			1	2	3	4	5	Average	Median	Mean deviation	standard deviation
S1	Blue value (g/100g)		1,12	1,16	1,1	1,1	1,12	1,12	1,12	0,0114	0,3670
	sand equivalent (%)		51	47	52	51	49	50	50,5	1,20	16,22
	Atterberg limit (%)	WL	40	41	43	39	42	41	41	0,86	13,32
		WP	27	27	26	25	25	26	26	0,57	8,44
		PI	13	14	17	14	17	15	14,5	1,20	4,73
S2	Blue value (g/100g)		0,06	0,08	0,12	0,08	0,06	0,08	0,08	0,0114	0,0285
	sand equivalent (%)		92	86	84	85	89	87,2	86,6	1,96	28,26
	Atterberg limit (%)	WL	Sandy soil								
		WP									
		PI									

Finally, in Table 4 we expose Chemical and mineralogical characteristics of sediment S1 and S2 dredged in Safi harbour.

**Table 4:** Chemical and mineralogical characteristic of sediment S1 and S2 dredged in Safi harbour.

Samples			1	2	3	4	5	Average	Median	Mean deviation	standard deviation
S1	Carbonate content %		90,5	92,5	95	95	93	**93**	93	1,44	1,69
	Organic content (%)	By chemical method	4,5	4	6	4,5	6	**5**	4,5	0,8	0,84
		By calcination method	7	6	7,8	6,2	9	**7,2**	7	0,96	1,10
	Mineralogical characteristic	Main phases	**Calcite quartz and Dolomite**								
		Minority phases	**Muscovite and clinochlore**								
S2	Carbonate content (%)		89	90	88,5	92,5	90	**90**	90	1	1,38
	Organic content (%)	By chemical method	0	0	0	0	0	**0**	0	0	0,00
		By calcination method	0,4	2,4	0,4	0,8	1	**1**	0,8	0,56	0,74
	Mineralogical characteristic	Main phases	**calcite, quartz and Dolomite**								
		Minority phases	**Muscovite, clinochlore and bassanite**								

## 2 Experimental design, materials, and methods

### 2.1 Sampling

**Fig. 1:**
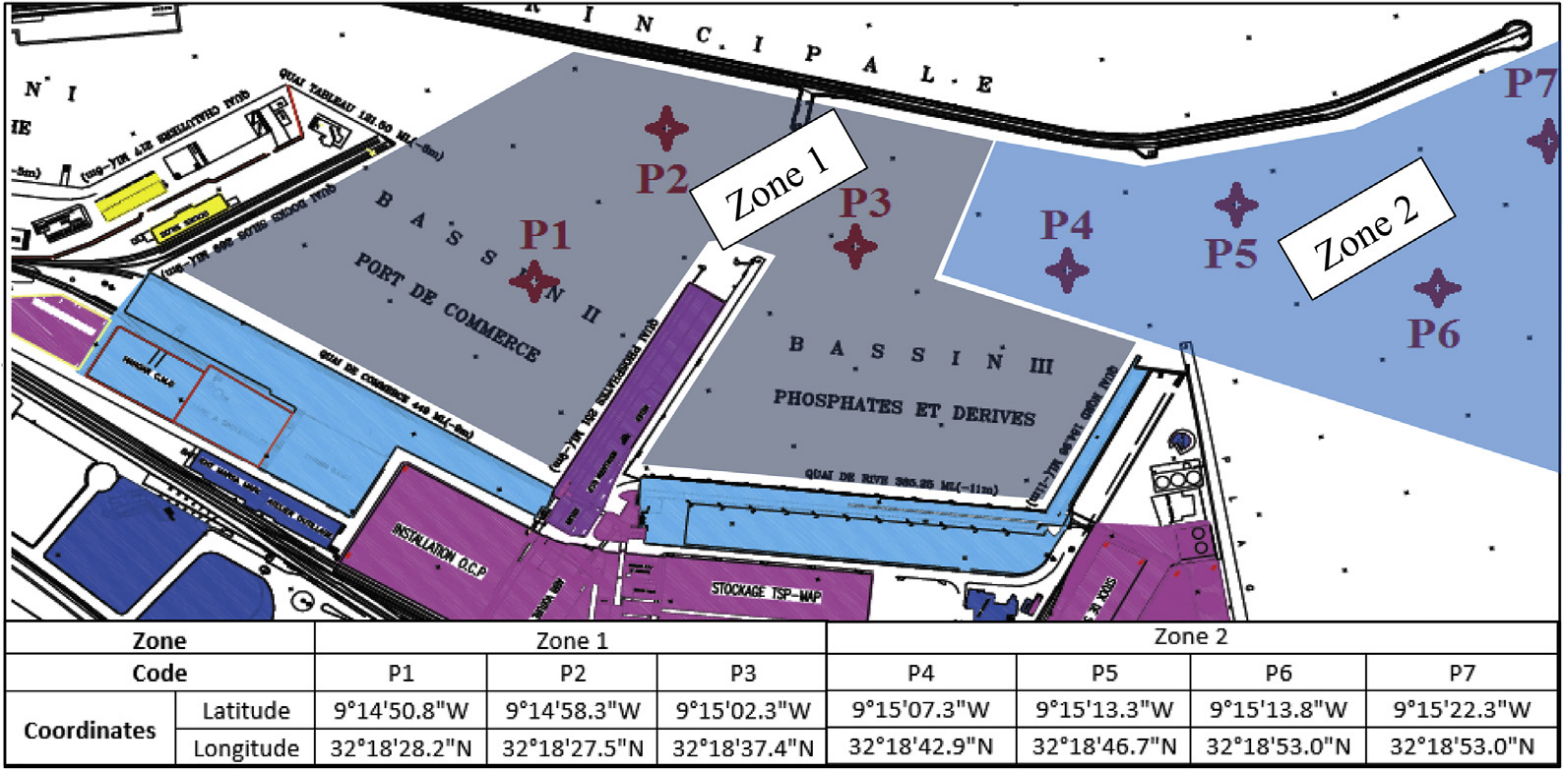
Coordinates of the sampling sites at Safi harbor.

The sampling is a crucial step in the process of characterization of a sediment. The goal is to obtain representative samples that reflects all the characteristics of the site. Sampling concerns two areas of the port: zone 1 and zone 2 which correspond respectively to the channel of access and the basins of the port of trade. Samples are collected by a diver and placed in opaque 20 Littre drums and then they are kept in a cold room protected from light. On each container, we mark the following information: The identification code of the sampling station, the date, the time and depth. In a first step, sediment samples were collected in 7 points (4 points in zone 1 and 3 point in zone 2). Then the samples were homogenized manually in order to have a single representative sample for each zone. We note S1 for the mixture of P1, P2 and P3 corresponding to the zone 1 and S2 for the mixture between P4, P5, P6 and P7 corresponding to the zone 2 (see Fig. 1).

### 2.2 Geotechnical characterization

The determination of the geotechnical characteristics of sediments consists in measuring the values of the water content, the specific density, the Atterberg limits, the sand equivalent, the methylene blue and the grain size distribution.

Measurement of the water content of a soil is the most common action in geotechnical and the most fundamental allows expressing many other values by bringing it to an expression of dry mass [1]. In our measures, we used the French standard NF P 94-050.

The particle-size-distribution analysis is used to determine the size of grain and the granularity (dimensional distribution of grains) of an aggregate or a soil. The aggregates are split by passing through different sieves up to 80 μm ac-cording to the French standard NF P 94-056. Below this dimension, the analysis is done by sedimentation by applying the instructions of the standard NF P 94-057.

The density (defined by the ratio between the mass of a sample dried in oven and the volume that it occupies in the water) is measured following the AFNOR standard NF P 94-054.

The determination of the Atterberg limits allows us to determine the plasticity index PI and the consistency index CI. The test is carried out in accordance with the standard NF P 94-051], it is applied on soils moderately to very clayey. For this reason, the determination of the “cleanliness” by Atterberg limits will be realized only on the sample S1.

The value of the methylene blue is the second parameter to characterize the “cleanliness” of a soil. His goal is to evaluate overall clay richness of the soil; This measurement uses the adsorption properties that the clay particles are nearly the only ones to possess in the soil, the fact that their surfaces are electrically charged, they adsorb a quantity of methylene blue proportional to the available surfaces when they are put in the presence of a solution of this dye (methylene blue). The test is realized by following the standard NF P 94-068.

The third parameter used to characterize the “cleanliness” of a soil is the sand equivalent.

### 2.3 Chemical and mineralogical characterization

The determination of the mineralogical and chemical characteristics allows us to understand the interactions between the sediment and the materials mixed with [2].

We had measured the content of organic matter and calcium carbonate and we had carried out a mineralogical analysis.

The determination of the organic content in sediment is an important step for the identification of characteristics and the track of valorisation. In effect, the organic matter is a component undesirable in a material of construction. We will use two methods to calculate the organic content of the sediment: the chemical method and the calcination method according to AFNOR standards NF P 94-055 and XP P 94-047 respectively [3,4].

The analysis of the content of calcium carbonate has been realized according to the principles of the standard NF P 94-048.

The mineralogical analyses are performed using the X-ray powders diffraction technique.
